# Can a morphological description of the peritoneal carcinomatosis in advanced ovarian cancer add prognostic information? Analysis of 1686 patients of the tumor bank ovarian cancer

**DOI:** 10.3389/fonc.2022.1014073

**Published:** 2022-12-21

**Authors:** Sara Nasser, Aygun Babayeva, Ioana Braicu, Rolf Richter, Esra Bilir, Radoslav Chekerov, Mustafa-Zelal Muallem, Klaus Pietzner, Melissa-Guelhan Inci, Jalid Sehouli

**Affiliations:** ^1^ Department of Gynecology with Center for Oncological Surgery, Charité–Universitätsmedizin Berlin, Virchow Campus Clinic, Charité Medical University, Berlin, Germany; ^2^ Department of Obstetrics and Gynecology, Stanford University, Stanford, CA, United States; ^3^ Department of Global Health, Graduate School of Health Sciences, Koç University, Istanbul, Turkey

**Keywords:** ovarian cancer, peritoneal carcinoma, peritoneal carcinomatosis index (PCI), morphology, survival outcome, ascites, intraoperative period

## Abstract

**Background:**

Peritoneal carcinomatosis in ovarian cancer is frequent and generally associated with higher stage and poorer outcome. The clinical features of peritoneal carcinomatosis are diverse and their relevance for surgical and long-term outcome remains unclear. We conducted this prospective study to describe intraoperatively the different features of peritoneal carcinomatosis(PC) and correlate them with clinicopathological features, progression-free(PFS) and overall survival (OS),.

**Methods:**

We performed a systematic analysis of all patients with documented intraoperative PC and a primary diagnosis of epithelial ovarian, tubal, or peritoneal cancer from January 2001 to September 2018. All data were evaluated by using the systematic tumor bank tool. Specific PC features included texture(soft-hard), consistency(coarse-fine or both), wet vs dry(PC with ascites vs. PC without ascites), and localization(diffuse-local). PC characteristics were then evaluated for correlation with age, FIGO-stage, histology, lymph-node involvement, grade, and presence of residual tumor at primary surgery. Moreover, the influence of PC characteristics on OS and PFS was analyzed.

**Results:**

A total of 1686 patients with PC and primary epithelial ovarian cancer were included. Majority of the patients were characterized by diffuse PC(73.9%). The majority of peritoneal nodules were fine in texture (55.3%) and hard in consistency (87.4%). Moreover, 27.6% of patients had dry PC. Diffuse PC localization was significantly associated with higher FIGO-stage (p<0.001), high-grade (p=0.003) and serous tumors (p=0.006) as well as residual tumor as compared to local PC (p<0.001). Wet PC also significantly correlated with diffuse localization (p <0.001) and residual tumor as compared to dry PC (p<0.001). Coarse PC was significantly associated with residual tumor as compared to fine PC (p=0.044). All other PC features didn´t correlate with clinicopathological features. As for survival outcomes, diffuse peritoneal localization (p<0.001), wet PC (p<0.001), and additional lymph node involvement (p<0.001) were associated with lower OS and PFS rates. Other PC features did not significantly impact survival.

**Conclusion:**

Diffuse localization of peritoneal carcinomatosis was significant predictor of recurrence. Lower OS and PFS were associated with diffuse peritoneal localization, wet PC, and additional lymph node involvement. Further prospective trials are warranted with the inclusion of translational research aspects to better understand the different peritoneal carcinomatosis patterns.

## Introduction

Among gynecologic malignancies ovarian cancer is the second most common and the number one cause of death (1). There were over 313,000 new ovarian cancer cases in 2020 worldwide (2). Symptoms for ovarian cancer are nonspecific, causing it to be diagnosed at advanced stages when tumor cells have already disseminated into the peritoneal cavity and to other organs. Intraperitoneal spread of ovarian cancer is the most typical, and the earliest distribution pathway (3, 4). Extent of intraperitoneal tumor spread determines International Federation of Gynaecology and Obstetrics (FIGO) stage and impacts residual mass after surgery, which is of big importance for prognosis (5, 6).

Clinical studies have shown that up to 50% of patients with infiltrations of the serosa develop peritoneal carcinomatosis (7, 8). Peritoneal carcinomatosis index (PCI) has been assessed as a powerful tool to describe peritoneal carcinomatosis (PC) in colorectal and gastrointestinal cancers. However, PCI focuses on size and distribution of peritoneal carcinomatosis and does not include specific characteristics of PC such as consistency and texture. Few studies have also evaluated PCI in ovarian cancer patients (13) with no effect on survival outcomes. Similarly, there was no inclusion of specific PC characteristics other than size and distribution (7–10). Hence, the role of PC and its diverse characteristics in complete tumor resection have not yet been sufficiently investigated, and their impact on survival remains controversial in patients with ovarian cancer (11–14).

In this study, our primary goal was to evaluate specific characteristics of PC (wet vs dry, fine vs coarse, hard vs soft, and local vs. diffuse) in patients with primary epithelial ovarian cancer (EOC), tubal cancer, and peritoneal cancer and also reveal their correlations with clinicopathological factors and residual tumor. Our secondary goal is to investigate their impact on progression-free survival (PFS) and overall survival (OS).

## Materials and methods

We performed a systematic analysis of all the patients operated at our tertiary center with documented intraoperative PC and a primary diagnosis of epithelial ovarian, tubal, or peritoneal cancer registered in the Tumor Bank Ovarian Cancer Network (TOC) database (15), an international prospectively maintained database, from January 2001 to September 2018. The local ethics commission granted the approval (No. EK207/2003), and all participating patients were well informed and signed the informed consent form before being included in the TOC database.

All the patients’ data included in the TOC database were documented using a validated mapping system known as the Intraoperative Mapping of Ovarian Cancer (IMO), which was developed for ovarian neoplasms with special focus on the description of the tumor pattern, maximal tumor-burden, and postoperative tumor-residuals (16). Briefly, IMO has three levels where one, two, and three were referred as lower, middle, and upper, respectively (16).

The spread of PC, presence of ascites, the residual tumor mass, and its localization were documented prospectively during debulking surgery *via* an interview with the surgeon. All patients underwent surgery in a single tertiary referral comprehensive cancer center. Each surgery was aimed to accomplish maximum tumor debulking (to no visible tumor left) and was performed *via* median laparotomy. The TOC database data on FIGO staging were updated based on the new classification by further subclassifying Stage III as IIIA, IIIB, and IIIC (17).

For this study, all data were retrospectively evaluated and checked for plausibility and completeness by the authors. Periodical patient follow-ups were performed to keep the database updated.

Based on the histopathological and surgical reports, PC was evaluated against the following characteristics based on known descriptive features for PC:

1. Presence of ascites: wet PC (presence of ascites) vs. dry PC (no ascites)
2. Texture of PC nodules: fine vs. coarse vs. both
3. Consistency of PC nodules: hard vs. soft
4. Localization of PC: local vs. diffuse

For the dissemination of PC, the term “local PC” was defined as PC limited to IMO Level 1, whereas “diffuse PC” was defined as covering more than IMO Level 1. For the term “Wet PC” any amount of ascites was included. Moreover, the ascites volume was subclassified as below or above 500 ml. Moreover the novel morphological characteristics of PC (consistency and texture) were defined by the primary surgeon upon palpation and inspection of the PC and documented in the IMO-mapping system.

The above PC characteristics were then evaluated for correlation with age of patients, FIGO stage at the time of diagnosis, histological tumor type, lymph node involvement, tumor grade, and presence of residual tumor at primary surgery(defined as any tumor size of ≥0.5cm) Moreover, the influence of PC characteristics on OS and PFS was also analyzed.

### Statistical analysis

Patient demographics and clinical characteristics were expressed as frequencies (%) for nominal data, as median values [interquartile range (IQR)] for continuous and non-normally distributed data, and as median with standard deviation (SD) for continuous and normally distributed data. Throughout the analysis, we calculated percentage values over the total number of eligible patients. Associations between the PC characteristics and clinical factors were analyzed using the χ^2^ test, Kendall’s tau b, Mann–Whitney U test, Kruskal–Wallis H test, or Spearman’s rho. Univariate and multivariate survival analyses was performed using the Kaplan–Meier method (log-rank testing) and Cox regression models. Moreover, hazard ratios (HR) and calculated in 95% confidence interval (CI) were calculated. Statistical analysis was performed using IBM SPSS Statistics (Version 21.0; IBM, Armonk, NY, USA). For all the tests, a probability value of p < 0.05 was considered statistically significant.

## Results

### Patients’ characteristics

A total of 1686 patients with primary diagnosis of ovarian cancer, tubal cancer, and peritoneal cancer were extracted from the TOC database from January 2001 to September 2018. Of the identified sample, 504 patients were excluded as they did not show intraoperative peritoneal carcinomatosis. Hence, our final sample included 1182 patients. Majority of the patients (90.7%) had ovarian cancer (**Table 1**).

**Table 1:** Clinical characteristics of the patients (n=1182).

Variable	Number (%)
Age (years)	60 (69 – 51)*
Tumor type	
Ovarian cancer	1072 (90.7)
Tubal cancer	30 (2.5)
Peritoneal cancer	80 (6.8)
FIGO stage	
II	37 (3.1)
III	761 (64.4)
IIIA	18 (1.5)
IIIB	48 (4.06)
IIIC	687 (58.1)
Missing	8 (0.7)
IV	299 (25.3)
Missing	85 (7.2)
Histology	
Serous	1044 (88.3)
Mucinous	23 (1.9)
Endometroid	32 (2.7)
Clear cell	11 (0.9)
Mixed tumors	10 (0.8)
Missing	62 (5.2)
Grade	
I	69 (5.8)
II	218 (18.4)
III	831 (70.3)
Missing data	64 (5.4)
Lymph node involvement	
N0	302 (25.5)
N1	616 (52.1)
Nx	221 (18.7)
Missing	43 (3.6)
Metastasis	
M0	330 (27.9)
M1	243 (20.6)
Mx	384 (32.5)
Missing	225 (19.0)
Residual tumor	
Macroscopically tumor free	710 (60.1)
< 0.5 cm	167 (14.1)
< 1 cm	141 (11.9)
< 2 cm	31 (2.6)
>2 cm	117 (9.9)
Missing	16 (1.4)

FIGO, International Federation of Gynaecology and Obstetrics.

*Interquartile range

The patients’ median age at initial diagnosis was 60 years (69 – 51). At primary diagnosis, the majority of patients had advanced FIGO stage III (64.4%) and IV (25.3%), respectively. Serous tumors were the most prevalent across the entire sample (88.3%). Among the studied sample, advanced grade III at initial diagnosis was the most common (70.3%). Positive lymph node involvement was documented in 52.1%. **Table 1** describes the clinical characteristics of the studied sample. Of the included sample, 716 patients underwent bowel resection where 57.9% and 22.9% were large bowel resection and small bowel resection, respectively.

### Characteristics of peritoneal carcinomatosis

On primary diagnosis, more than half of the patients were characterized by diffuse localization of peritoneal carcinomatosis (62.4%). The majority of peritoneal nodules were described as fine in texture (47.5%) and hard in consistency (59.6%). Majority of the patients (71.7%) had wet peritoneal carcinomatosis. **Table 2** demonstrates the distribution of the characteristics of peritoneal nodules.

**Table 2:** Characteristics of peritoneal carcinomatosis (n= 1182).

Variable	Number (%)
Localization	
Local	260 (22.0)
Diffuse	738 (62.4)
Missing	184 (15.6)
Consistency	
Hard	705 (59.6)
Soft	102 (8.6)
Missing	375 (31.7)
Texture	
Fine	562 (47.5)
Coarse	366 (31.0)
Both	88 (7.5)
Missing	166 (14.0)
Presence of ascites	
No ascites(Dry PC*)	323 (27.3)
Ascites(Wet PC)	847 (71.7)
< 500 ml	395 (33.4)
> 500 ml	452 (38.2)
Missing	12 (1.0)

PC, Peritoneal carcinomatosis.

### Correlation of characteristics of peritoneal carcinomatosis and clinicopathological features

Age at initial diagnosis was significantly higher in patients with diffuse PC (p < 0.001). Moreover, FIGO staging significantly correlated with diffuse PC (p < 0.001) and wet peritoneal carcinomatosis (p < 0.001). Serous histology was significantly associated with diffuse PC (p = 0.006). Tumor grade was significantly associated with diffuse PC (p = 0.003) and wet peritoneal carcinomatosis (p < 0.001). Diffuse PC significantly correlated with presence of residual tumor as compared to local PC (p = 0.001). Similarly, coarse PC correlated significantly with presence of residual tumor as compared to fine PC (p = 0.044). Wet PC also significantly correlated with residual tumor as compared to dry PC (p < 0.001).

Tumor localization as characterized according to IMO criteria, displayed no statistically significant associations with any of the characteristics associated with peritoneal carcinomatosis (consistency, p = 0.799; texture, p = 0.464; and ascites, p = 0.069). **Table 3** showcases the correlations between different characteristics and peritoneal carcinomatosis. Moreover, positive lymph node involvement with presence of PC was significantly associated with residual tumor (p < 0.001). However, it did not significantly correlate with any PC specific morphological characteristics. The number of patients with PC and without residual tumor who had N0 and N1 were 231 (19.5%) and 390 (33.0%), respectively. The majority of the patients with PC and residual tumor (194, 80.2%) actually had positive lymph node involvement(N1).

**Table 3:** Correlation of peritoneal carcinomatosis characteristics with clinicopathological features (n=1182).

Clinicopathological Features	Characteristics of peritoneal carcinomatosis (%)												
	Localization of PC			Texture of PC nodules				Consistency of PC nodules			Presence of Ascites		
	Local	Diffuse	p-value	Fine	Coarse	Both	p-value	Hard	Soft	p-value	Wet	Dry	p-value
**Histology**													
Serous	220 (18.6)	665 (56.3)	**0.006**	499 (42.2)	323 (27.3)		0.257	631 (53.3)	91 (91.9)	0.505	764 (64.6)	270 (22.8)	0.341
Non-serous	26 (2.2)	38 (3.2)		36 (3.1)	25 (2.1)			41 (3.5)	8 (8.1)		55 (4.7)	21 (1.8)	
**FIGO Stage**													
II	23 (1.9)	6 (0.5)	**<0.001**	17 (1.4)	5 (0.4)		0.079	18 (1.5)	4 (4.2)	0.112	20 (1.7)	17 (1.4)	**<0.001**
III	173 (14.6)	472 (39.9)		380 (32.1)	228 (19.3)			450 (38.1)	70 (73.7)		543 (45.9)	212 (17.9)	
IV	48 (4.1)	213 (18.0)		131 (11.1)	110 (9.3)			192 (16.2)	21 (22.1)		226 (19.1)	68 (5.8)	
**Grading**													
G1	24 (2.3)	31 (2.6)	**0.003**	30 (2.5)	21 (1.8)		0.941	37 (3.1)	9 (9.1)	0.126	36 (3.0)	33 (2.8)	**<0.001**
G2+G3	222 (18.8)	665 (56.3)		508 (43.0)	323 (27.3)			629 (94.4)	90 (90.9)		778 (65.8)	261 (22.1)	
**Residual Tumor**													
TR=0	226 (37.7)	373 (31.6)	**0.001**	347 (29.4)	207 (17.5)		**0.044**	404 (34.2)	65 (5.5)	0.235	467 (39.5)	238 (20.1)	**<0.001**
TR(≥ 0.5 cm)	31 (2.6)	358 (30.3)		210 (17.8)	155 (13.1)			296 (25.0)	36 (3.0)		372 (31.5)	81 (6.9)	
**IMO Level**													
1	NA	NA	NA	541 (45.8)	353 (29.9)		0.464	684 (57.9)	99 (8.4)	0.799	820 (69.4)	302 (25.5)	0.069
2	NA	NA		462 (39.1)	333 (28.1)			634 (53.6)	83 (7.0)		753 (63.7)	246 (20.8)	
3	NA	NA		392 (33.2)	296 (25.0)			553 (46.8)	74 (6.2)		662 (56.0)	191 (16.1)	

IMO, Intraoperative Mapping of Ovarian Cancer.

TR, tumor residue.

The bold p-values indicated statistically significant result.

NA, not applicable.

The bold p-values indicated statistically significant result.

FIGO, International Federation of Gynaecology and Obstetrics.

Additionally, when looking at the group of low-grade tumors (G1) (n=75), the majority were diffuse in localization (56.3%), wet (52.1%), hard in consistency (80.4%), and fine in texture (53.6%). These features were similar in the group of high-grade tumors (G2 and G3). However, tumor grading did not correlate significantly with any of the above morphological PC features.

When examining the correlation between residual tumor presence and PC characteristics stratified by IMO level of PC involvement, residual tumor was significantly associated with wet PC at all three levels of involvement (all, p <0.001). The other features did not correlate significantly.

### Correlation of peritoneal carcinomatosis characteristics with survival outcomes

**Figure 1:**
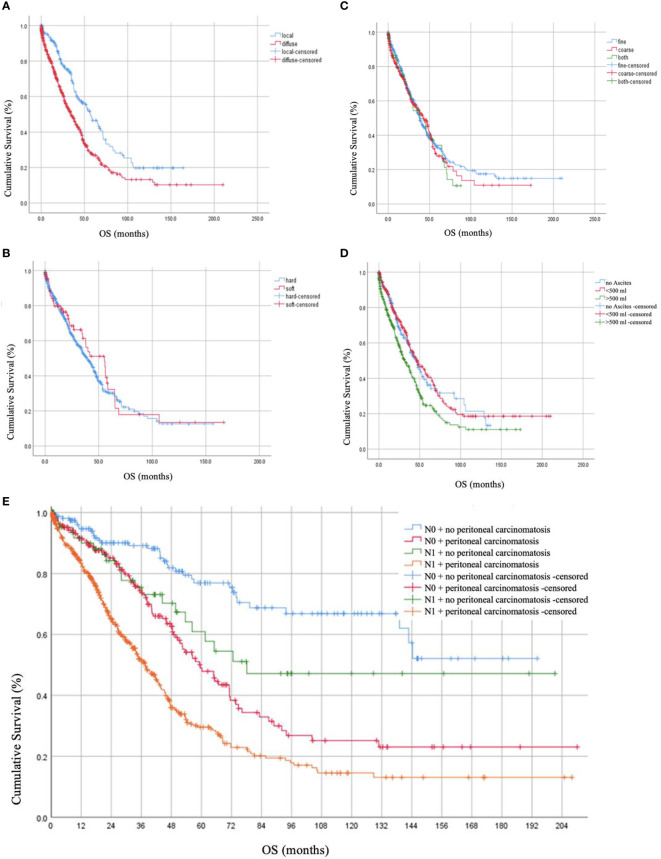
Overall Survival (OS) rates in months based on **(A)** localization of peritoneal carcinomatosis, **(B)** consistency of peritoneal carcinomatosis, **(C)** texture of peritoneal carcinomatosis nodules, **(D)** presence of ascites together with peritoneal carcinomatosis, and **(E)** lymph node involvement.

**Figure 2:**
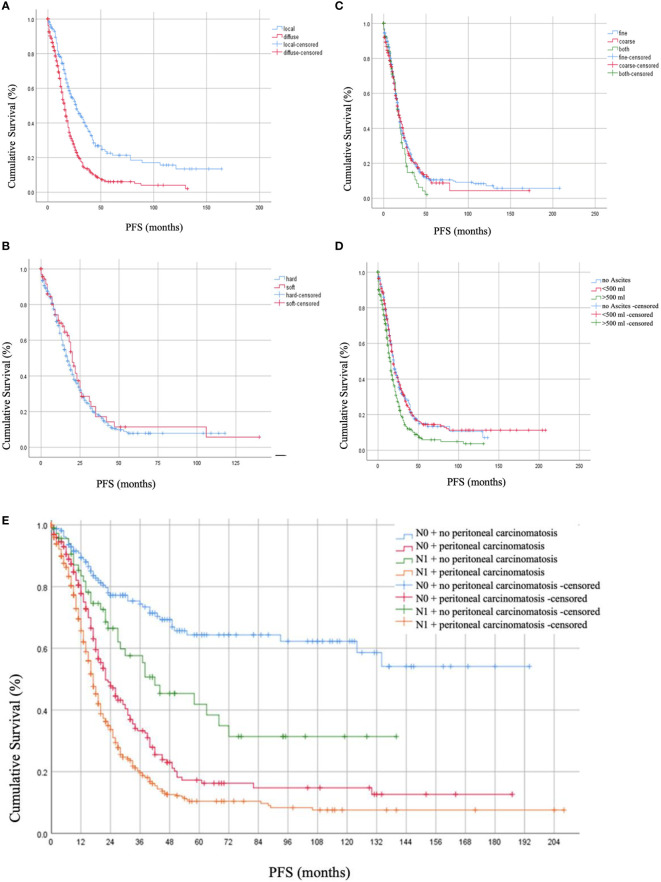
Progression-free Survival (PFS) rates in months based on **(A)** localization of peritoneal carcinomatosis, **(B)** consistency of peritoneal carcinomatosis, **(C)** texture of peritoneal carcinomatosis nodules, **(D)** presence of ascites together with peritoneal carcinomatosis, and **(E)** lymph node involvement.

Post-operative residual tumor was present in 39.1% of the patients (**Table 1**). The studied cohort was followed up for a median of 17.0 months (Range: 1.8 - 37.8 months), throughout which, 41.2% presented with recurrence while 42.6% died due to the disease. Using log-rank testing, diffuse peritoneal localization (p < 0.001), wet peritoneal carcinomatosis with ascites more than 500 ml (p < 0.001), and additional lymph node involvement (p < 0.001) were associated with lower OS rates (**Figure 1**). However, when using a multivariate cox-regression model, age at initial diagnosis (HR: 1.019; 95% CI: 1.008–1.030; p = 0.001), positive lymph node involvement (HR: 1.679; 95% CI: 1.295–2.176; p < 0.001), and presence of residual tumor (HR: 1.709; 95% CI: 1.345 – 2.171; p < 0.001) were found to be predictors of lower OS due to the peritoneal carcinomatosis. Similarly, on univariate log-testing, peritoneal diffuse localization (p < 0.001), wet PC with presence of ascites more than 500 ml (p < 0.001), and additional lymph node involvement (p < 0.001) were associated with lower PFS rates (**Figure 2**). Nonetheless, on multivariate cox-regression, age at initial diagnosis (HR: 1.010; 95% CI: 1.001 – 1.019; p = 0.025), diffuse peritoneal dissemination (HR: 1.567; 95% CI: 1.250 – 1.965; p < 0.001), positive lymph node involvement (HR: 1.310; 95% CI: 1.044 – 1.642; p = 0.019), and presence of tumor residue (HR: 1.335; 95% CI: 1.078 – 1.653; p = 0.008) were associated with lower PFS due to peritoneal carcinomatosis (**Table 4**).

**Table 4:** Correlation of peritoneal carcinomatosis characteristics with survival outcomes.

	Overall Survival			
	p-value*		HR (95% CI)	CI (95%)
Age at diagnosis	**0.001**		1.019	1.008-1.030
Ascites >500 ml	0.107		1.218	0.958-1.547
Diffuse peritoneal dissemination	0.129		1.229	0.942-1.603
Lymph node involvement	**<0.001**		1.679	1.295-2.176
Residual tumor after primary surgery	**<0.001**		1.709	1.345-2.171
	**Progression-free Survival**			
	p-value*	HR (95% CI)		CI (95%)
Age at diagnosis	**0.025**	1.010		1.001-1.019
FIGO	0.061			
III	0.472	1.306		0.631-2.706
IV	0.177	1.679		0.791-3.561
Diffuse peritoneal dissemination	**<0.001**	1.567		1.250-1.965
Lymph node involvement	**0.019**	1.310		1.044-1.642
Residual tumor after primary surgery	**0.008**	1.335		1.078-1.653

CI, confidence interval.

FIGO, International Federation of Gynaecology and Obstetrics.

HR, hazards ratio.

*The bold values indicated statistically significant result.

### Survival predictors by grade

Across the high-grade tumors (G2 and G3 tumors), age at diagnosis (HR: 1.020; 95% CI: 1.009 – 1.031; p < 0.001), positive lymph node involvement (HR: 1.606; 95% CI: 1.244 – 2.073; p < 0.001), and presence of residual tumor (HR: 1.652; 95% CI: 1.310 – 2.082; p < 0.001) were significantly associated with lower OS. However, only diffuse peritoneal involvement (HR: 1.511; 95% CI: 1.189 – 1.921; p = 0.001), and residual tumor presence (HR: 1.320; 95% CI: 1.061 – 1.643; p = 0.013) were significantly associated with lower PFS across the studied sample of high-grade tumors (n=1107)

In the subgroup of low-grade tumors (G1) (n=75, 6.3%) lower OS was significantly associated with age at diagnosis (HR: 1.059; 95% CI: 1.018 – 1.101; p = 0.004), and residual tumor (HR: 2.856; 95% CI: 1.143 – 7.138; p = 0.025). On the other hand, when analyzing PFS, only residual tumor was significantly associated with lower PFS rates amidst low-grade tumors (HR: 3.166; 95% CI: 1.462 – 6.857; p = 0.003).

## Discussion

To our knowledge, this is the only study describing the specific characteristics (beyond localization and presence of ascites) of peritoneal carcinomatosis in patients with ovarian cancer prospectively. Most description of PC have been reported in gastric and appendiceal tumors (18, 19). However, even here the focus has remained on describing localization and presence of ascites (19–22).

Indeed diffuse localization of peritoneal carcinomatosis was a significant predictor of recurrence in our multivariate model. This is a finding well documented within the literature (18). The majority of peritoneal carcinomatosis in our cohort was hard in consistency, wet (i.e., ascites producing), and fine in texture.

There have been very few reports on descriptive morphological features of peritoneal carcinomatosis in patients with EOC. In one retrospective observational study, the authors aim to describe texture and consistency features of appendiceal tumors with peritoneal metastases (19). Features such as hard consistency significantly correlated with lower survival outcome as compared to tumors that were described as soft in consistency (19, 20).

Our study demonstrated that coarse texture of peritoneal carcinomatosis was more significantly associated with residual tumor regardless of localization. This, however, did not influence survival in our study. However, so far specific description or correlation of coarse PC in other tumors is very sparse in the literature. In another histopathological analysis of appendiceal tumors with peritoneal metastasis, the authors demonstrated that hard and coarse peritoneal carcinomatosis are associated with lower overall survival (19). The authors describe the theory that hard tumors produce a sialic acid rich mucin that may act as a protective barrier to cancer cells as it can inhibit the efficacy of chemotherapeutic agents (19).

In our study, consistency and texture of peritoneal carcinomatosis did not correlate with survival or overall prognosis. However, our results demonstrate that specific morphological features of peritoneal carcinomatosis, while not necessarily predictive of survival, were associated with more aggressive clinical tumor characteristics.

Furthermore, our results reinforce that diffuse localization of PC, large volume ascites (> 500 ml), and additional lymph node involvement were significantly associated with lower median OS and PFS. Diffuse dissemination was also a predictor of lower PFS rates on multivariate regression. Interestingly, when looking at tumor grading it appeared that diffuse peritoneal localization is a negative predictor of PFS in high-grade tumors(G2-G3) and not in low-grade tumors(G1). Although the subgroup sample size of low-grade tumors in this cohort was too small for the result to be statistically significant this does warrant future investigation.

Additional nodal involvement with the presence of PC was associated with worse survival outcomes both in OS and PFS in our data. This is compatible to what is known in the literature (23–26). Lymphatic spread has been reported to be a common feature and prognostic factor in both early and advanced stage ovarian cancer (27, 28). Reports demonstrate that diffuse peritoneal disease was associated with involvement of pelvic lymph nodes (25). It appears that the establishment of peritoneal disease is more important in the development of nodal disease than even primary tumor characteristics in advanced ovarian cancer (24). This was further established in the prospective study by Harter et al. on lymph node dissection in EOC patients which showed no benefit in survival for systematic lymph node dissection in patients with advanced stage EOC (27). Although this assessment was outside of the scope of our study, it is an interesting observation that warrants further investigation.

Our study has several limitations. We included patients across different FIGO stages, histology, and grades. Thus, further studies are warranted to investigate the role of morphological descriptive PC classification in overall survival among patients with ovarian cancer in specific subgroups including the use of maintenance therapies, such as PARP-inhibitors and/or bevacizumab. Although we extracted data from a prospectively maintained database, we had missing data on various variables which were clearly presented in **Tables 1**, **2**. Moreover, it is noteworthy that consistency and texture are two subjectively evaluated morphological features which may increase interobserver bias. However, these features have been systematically documented over a period of more than twenty years as part of the IMO-system. Our median follow-up was 17.0 months. Thus, future studies with longer follow-up period to investigate the impact of morphological descriptive PC classification on the survival outcomes are needed.

On the other hand, our study also has numerous strengths. Firstly, we evaluated intraoperative PC features exclusively in primary ovarian cancer patients where the literature has been dominated by other cancer types. The focus on specific morphological characteristics of PC, in addition to the known size and distribution patterns, has rarely been described. Moreover, since we investigated the intraoperative PC features, our results are applicable to daily clinical practice in high-income countries as well as in low- and middle-income countries

In conclusion, our study demonstrates that diffuse localization of peritoneal carcinomatosis was a significant predictor of recurrence and that coarse texture was significantly associated with residual tumor at primary surgery. These results support the mandatory incorporation of describing the morphological features of peritoneal carcinomatosis in ovarian cancer. Other specific characteristics of peritoneal carcinomatosis, while not necessarily predictive of survival, are associated with more aggressive clinical tumor characteristics. Further prospective trials are warranted with the inclusion of translational research aspects to better understand the different peritoneal carcinomatosis patterns and morphological features.

## Data availability statement

The raw data supporting the conclusions of this article will be made available by the authors, without undue reservation.

## Author contributions

SN and JS: Designing of study concept, organizing and execution. Data review, and writing of manuscript. AB and EB: Data collection, Data Analysis, Review of manuscript. IB: Study design, Data Analysis and Review of manuscript. RR: Statistical design, Data Analysis, Review of manuscript. M-ZM, M-GI, KP, and RC: Data Analysis, major manuscript revisions. All authors contributed to the article and approved the submitted version.
